# Language and imagined *Gesellschaft*: Émile Durkheim’s civil-linguistic nationalism and the consequences of universal human ideals

**DOI:** 10.1007/s11186-020-09394-1

**Published:** 2020-05-04

**Authors:** Mitsuhiro Tada

**Affiliations:** Faculty of Humanities and Social Sciences, Kumamoto University, 40-1, Kurokami 2-chome, Chuo-ku, Kumamoto, 860-8555 Japan

**Keywords:** Civil religion, Laic education, Linguistic turn, National society, Regional language, Sociology of knowledge

## Abstract

When Thomas Luckmann, a pioneer of the “linguistic turn” in sociology, regarded Émile Durkheim as a source for the sociology of language, he had lifeworldly community–building in mind. However, the French sociologist himself understood language in the context of *civil society–building*. To Durkheim, language was a “social thing in the highest degree” that enabled general ideas and intermediated them to people. Abstract human ideals like the civil religion since the French Revolution could be shared through (a common) language. Thus, Durkheim took the exclusive use of French in the Third Republic’s laic public education for granted, ignoring the patois in the country: This “child of the Enlightenment” considered French to be a universal language of *Gesellschaft* and, beyond ethno-communal elements, to work as a basis for the organic solidarity of French national civil society where the social division of labor was progressing. Durkheim’s theory was predicated on civil-linguistic, not ethnolinguistic, nationalism.

*An honorable fact for France is that it has never sought to obtain the unity of the language by means of coercion. Can’t we have the same feelings and thoughts, love the same things in different languages?* […] *Let’s not abandon the fundamental principle that a human being is a rational and moral being before being cooped up in this or that language, before being a member of this or that race, an adherent of this or that culture. Before the French culture, the German culture, the Italian culture, there is the human culture.*

—From *What is a Nation?* (Renan [1882] 1887: 299, 300–301)

*Then, from one thing to another, Monsieur Hamel started talking to us about the French language, saying that it was the most beautiful language in the world, the most clear, the most solid: that we shall retain it between us and never forget it, because when a people falls into slavery, so long as it holds its language well, it is as if it held the key to its prison.*

—From *The Last Class: The Story of a Little Alsatian* (Daudet [1872] 1873: 23)

*The aborigines are going to be able to speak their patois, sorry, their language, without being laughed in the face.*

—From *Charlie Hebdo* (Oncle Bernard 1998, October 7: 16)

This article aims to clarify Émile Durkheim’s linguistic view and its implicit and peculiar methodological nationalism—I will characterize them generically as *civil-linguistic nationalism* below—referring to the social circumstances in France of his days that determined his view. Thomas Luckmann, a phenomenological sociologist of knowledge who led the “linguistic turn” in sociology in the mid-1960s, wrote in his 1975 *Sociology of Language*, looking back to the early 1960s before the turn, “At that time the sociology of language did not yet exist” (Luckmann 1975: 5); that is, the sociology of language had grown into a “new field” over only approximately ten years (Luckmann 1975: 5). Nevertheless, he first named Durkheim as a source for the sociology of language (Luckmann 1975: 13–14, 19). Indeed, it seems that this French sociologist had the most awareness of the social significance of language among the fathers of today’s sociology. Georg Simmel and Max Weber dealt with language rather peripherally, although they would have been surrounded by a social organicism linked with ethnolinguistic nationalism in Germany, as well as by the tradition of the philosophy of language that went back to Wilhelm von Humboldt (see Knoblauch 2003: 582).

Durkheim also stayed in Leipzig, Berlin, and Marburg for half a year in 1886 and might have been influenced by the German intellectual climate. However, language seems to have mattered to Durkheim within a peculiarly French background. To begin with, he was one of the promising young scholars sent to Germany by the Third Republic to learn from the victors of the 1870–1871 Franco-Prussian War (Tiryakian 1978, p. 193–194). Partly for this reason, Durkheim shared the Republic’s recognition of issues: For instance, the Republic had attributed its defeat to a lack of national awareness and thus had erected a primary-education system that would promote a strong French national identity (Nakamoto 2008, p. 34; see also Baubérot [2000] 2008, p. 42); Durkheim’s sociology was also directed toward creating “good Frenchmen” and moral unity among them.

Nor is his sociology of language an exception; in fact, language seems to underlie his whole social theory. However, despite his awareness of language’s importance, he never intensively developed linguistic sociology. Hence, in the following discussion, I will reconstruct his view of language from his fragmentary descriptions about language.^1^

First, I articulate the theoretical position of language for Durkheim. Less famously, he characterized language as a *social thing in the highest degree*, a fundamental social fact that enables ideal systems like religion, morality, laws, economy, and aesthetics and intermediates them to people. Second, I will show that Durkheim assumes the linguistic unity of France to be indispensable for social (and national) integration; French makes it possible for French citizens to share *civil religion*, a system of ideals, that Durkheim called *moral individualism* and that leads to organic solidarity despite the social division of labor. Third, I will also indicate that, as a prerequisite to sharing civil morality, Durkheim suggested the exclusive use of French in laic school education, although France in those days was a multilingual country. In his view, those who shared the Revolution’s modern human ideals beyond lifeworldly ethno-communal elements were free and equal members of French civil, yet national, society and, therefore, had to share the French language as a universal medium beyond patois. As I suggest above, one could name this view *civil-linguistic nationalism* to distinguish it from *ethnolinguistic nationalism* that, as seen in Germany, identifies the nation with a community in which the people ethnically share a language (and the *Volksgeist* based on it).

In the French Third Republic, French monolingualism was a presupposition for laic education to make all the people (in particular, all the children) a posteriori into French citizens who hold common civil-religious ideals or *society’s soul*. Durkheim’s view on language seems to have been closely associated with this national goal, underlying his sociology of education and that of religion, and it would therefore have to receive special attention. On the other hand, from a sociological perspective, it should not be overlooked that the self-identification of French national society as a “community of citizens” can, in this globalized era, lead to or be abused for exclusionism. In the conclusion, I will refer to this actual, paradoxical phenomenon of French modern human ideals instilled through a laic education—but similar phenomena may more or less be found in other national societies today.

## Language as a social thing in the highest degree

### Durkheim’s project of linguistic sociology

With the “linguistic turn” in sociology, language was deemed to be the collective macro-foundation for individuals’ mutual understanding, substituting for the religious value that Talcott Parsons supposed was shared as a facilitator of social integration. As Tada (2015) describes, while traditional (i.e., premodern) religion had been losing its normative communality through post-WWII industrialization and individualization, Luckmann newly declared language “a social a priori (*ein gesellschaftliches Apriori*)” (Luckmann 1983, p. 1573). In his view, language mediates between human beings, between human beings and objective reality, and between human beings and their sociocultural history (Luckmann [1973] 1983, p. 79). Luckmann thus saw language as a lifeworldly collective foundation given to individuals, saying, “the human kind of community-building (*Gemeinschaftsbildung*) without language is inconceivable” (Luckmann 1962, p. 514). By transmitting intersubjective knowledge, language joins individuals into a “historical community” (see Luckmann [1973] 1983, p. 90). In this context, he names Durkheim:

For an accurate description of the structure and function of sign-systems [i.e., human language], […] one should return to the original Durkheimian and de Saussurean view of sign-systems as *specific* systems of communication with a certain degree of “autonomy” with respect to other social systems of meaning. (Luckmann [1973] 1983, p. 73, emphasis in original)

This citation suggests a shift in the sociological interpretation of Durkheim, because Parsons’s theory of shared religious values also relied on Durkheim; after Parsons, Durkheimian collectivism resonated with phenomenological sociology of knowledge in terms of language.^2^ This shift is no contradiction. Durkheim himself remarks, “The system of concepts with which we think in everyday life is the one that the vocabulary of our mother tongue expresses; for every word translates a concept” (Durkheim 1912, p. 619 = [1915] 1964, p. 433), and therefore “In the word, there is […] condensed a whole knowledge (*science*) to which I have not collaborated, that is, a knowledge more than personal; and it surpasses me to such an extent that I cannot even completely appropriate all its results for myself” (Durkheim 1912, p. 621 = [1915] 1964, pp. 434–435). As detailed below, language for Durkheim is a *social fact*—a type of acting, thinking, and feeling external to an individual and vested with binding force (Durkheim [1895] 1956, pp. 4–5 = 1982, p. 51).

However, Durkheim’s concern with language did not always lie in the formation of lifeworldly community. Even as he seems to discuss community-building, *community* denotes the so-called “community of citizens (*communauté des citoyens*),” which Dominik Schnapper ([1994] 2003) characterizes as an ideal political society of free and equal citizens beyond objective-seeming elements like religion or ethnicity, connoting Republicanism. In other words, Durkheim’s community is that equated with French civil (and national) society. In fact, he himself described French society as a “communion of minds (*communion des esprits*)” (Durkheim [1898] 1987b, p. 272 = 1973b, p. 52), whose members were associated through respect for the abstract individual. Durkheim’s view of language must also be grasped in this context: He attended to *society-building* (*Gesellschaftsbildung, Vergesellschaftung*; societization or sociation) by citizens who shared modern human ideals and spoke French, the “common language of citizens for free communication” (see R. Balibar 1991, p. 74) above the vernaculars of lifeworldly communities.

By 1909 at the latest, Durkheim considered language to constitute its own sociological research field. He tentatively subclassified sociology into three special sciences: general sociology, social morphology, and social physiology. He then further subdivided social physiology, or science of the functions of social(−psychological) institutions based on collective lives, representations, and ideas, into six more specialized fields: religious sociology, moral sociology, legal sociology, economic sociology, aesthetic (artistic) sociology, and *linguistic sociology* (*sociologie linguistique*) (Durkheim [1909] 1987c, p. 149–153). These six divisions correspond to the specific *social facts*: religion, morality, laws, economy, aesthetics, and language, which consist of collective beliefs, customs, and institutions irreducible to individuals. As for the relevance of language to sociology, Durkheim remarks:

Language, which depends on organic conditions in certain respects, is, however, a social phenomenon: because it is also always the work of a group whose mark it carries. Language is, in general, even one of the elements characteristic of the appearance of societies, and it is not without reason that the affinity of languages is often used as a means to establish the kinship of the peoples. There is, therefore, a material for a sociological study of language, which has, furthermore, started. (Durkheim [1909] 1987c, p. 150)

Note the last sentence. A sociological study of language had been undertaken by other persons, and therefore Durkheim did not need to intensively develop linguistic sociology alone. An example is Antoine Meillet, whose article “How Words Change Meaning” (Meillet 1906) Durkheim referred to in the above passage, although, in reality, this French linguist had applied Durkheim’s notion of social fact to language, saying:

[I]t [language] enters exactly into the definition that Monsieur Durkheim has proposed; a language exists independently from each of the individuals who speak it and, although it has no reality apart from the sum of individuals in question, it however is, besides its generality, external to each of them: What it shows is that language does not depend on any of individuals in order to change, and all deviation in using it provokes a reaction. […] Therefore, the characteristics of externality to the individual and of coercion by which Durkheim defines the social fact appear in language with the greatest evidence. (Meillet 1906, pp. 1–2)

Durkheim thought that individuals should obey a pre-existing social fact; they could surely resist it, but they would inevitably be sanctioned by a collective hostility. Thus, Durkheim described a social fact as “objective” (Durkheim [1895] 1956, pp. 3–4 = 1982, pp. 50–51). Meillet encouraged other linguists to adopt this notion in their research.

Ferdinand de Saussure, who also studied at the Universities of Leipzig and of Berlin between 1876 and 1880, and whose class Meillet joined at École Pratique des Hautes Études in Paris, had a similar linguistic view to Durkheim: De Saussure stressed, while indicating the arbitrariness (contingency) of the bond between the signifier (*signifiant*) and the signified (*signifié*), that the bond has binding force for individuals. “The signifier, though appearing as freely chosen concerning the idea that it represents, is on the other hand fixed, not free, concerning the linguistic community that uses it. […] [T]hey [the masses] are bound to the language as it is” (De Saussure [1916] 1969, p. 104). Thus, the father of modern linguistics also called language a “social fact” or “social institution” (De Saussure [1916] 1969, p. 21, 33, 129) that binds individuals from outside and resists an arbitrary transformation by them.

Additionally, Marcel Mauss, Durkheim’s nephew and collaborator, recognized the social aspect of language. Durkheim had also hoped that his son, André, would study linguistics and even asked Meillet to train him, although the hopeful young linguist later died in World War I (see Tanabe 1981, pp. 34–35). In any case, since these other scholars had undertaken sociological investigation of language, Durkheim himself could do with fragmentary mention of language without systematizing his own linguistic sociology. At least, he refrained from the technical discussion of linguistics because of his lack of expertise (Durkheim 1912, p. 112 = [1915] 1964, p. 79).

However, another reason remains conceivable: Language was so fundamental that it worked as an unquestioned premise underlying his theory. He gives special status to language among the six social facts. “Language is,” he says, “*a social thing in the highest degree* [*chose sociale au premier chef*]; it is society which elaborated language, and it is society by which language is transmitted from generation to generation” (Durkheim [1925] 2012, p. 83 = [1961] 1973, p. 69, emphasis added). To my knowledge, Durkheim applies this characterization often, but only to language. Yet, he in no way clarified the reason for his privileging of language. Hence, I shall reconstruct his linguistic view from his scattered descriptions of language in terms of society-building.

### Language and general ideals

Durkheim indicates two functions of language in society: enabling human beings to acquire an inherited system of (abstract) ideas; and (thereby) bringing human beings into intellectual existence:

In learning a language, we learn a whole system of ideas, distinguished and classified, and we inherit all the work from which have come these classifications that summarize centuries of experiences. There is more: without language, we would not have, so to speak, general ideas; for it is the word which, in fixing them, gives to concepts a consistency sufficient for them to be able to be handled conveniently by the mind. It is, therefore, language which has allowed us to raise ourselves above pure sensation; and it is not necessary to demonstrate that language is, in the highest degree, a social thing. (Durkheim 1922, p. 57 = 1956 p.77)

This insight relates to Durkheim’s “sociological theory of knowledge (*théorie sociologique de la connaissance*)” (Durkheim 1912, p. 25 = [1915] 1964, p. 18; see also Tanabe 1981, chap. 4). According to him, a human being thinks with concepts, including cognitive categories like time, space, and causality, which Durkheim counts as social things irreducible to individual perceptions (Durkheim 1912, pp. 625–633 = [1915] 1964, pp. 438–444). While sensual representations of the individual continually change like a stream, concepts crystallize social manners of thinking, resisting change (see Durkheim 1912, p. 618 = [1915] 1964, p. 433). Therefore, to think conceptually is “to subsume the variable under the permanent, the individual under the social” (Durkheim 1912, p. 627 = [1915] 1964, p. 439).

Language appears here as the ultimate social thing. Durkheim argued that a purely personal concept is impossible. Unlike sensation, any idea is penetrated by “usˮ: “A concept is not my concept; I hold it in common with other people, or, in any case, can communicate it to them” (Durkheim 1912, p. 619 = [1915] 1964, p. 433, see also Durkheim [1914] 1987d, p. 318, note 1 = 1973b, p. 152, note 5). Durkheim then found in language the ground for such an impersonality (i.e., socialness) within mental life:

[C]oncepts are always common to a plurality of human beings. They are constituted by means of words; the vocabulary and grammar of a language are neither the work nor thing of one particular person; they are the product of a collective elaboration, and they express the anonymous collectivity that employs them. [...] I share it [a concept], to a large degree, with all the people who belong to the same social group that I do. Because of being in common, concepts are the supreme instrument of all intellectual exchange. By means of them, minds communicate. (Durkheim [1914] 1987d, pp. 317–318 = 1973b, pp. 151–152)

Based on this presupposition, Durkheim added that the abstraction of concepts is also due to language. General ideas that are immutable beyond instantaneous perceptions form only linguistically and, accordingly, human thought is always conditioned by language. “As Rousseau demonstrated long ago: deprived of all that comes from society, a man remains a being reduced to sensation, and a being more or less indistinct from an animal. Without language, a social thing in the highest degree, general or abstract ideas are practically impossible, as are all the higher mental functions” (Durkheim [1906] 1951c, p. 79 = 1965, p. 55, see also Durkheim 1912, p. 602 = [1915] 1964, p. 421).

In Durkheim’s view, language makes people into social beings with intellect. If this is correct, an abstract thought articulated by language would be more intersubjective than a sensation, just as a rational action is easier to understand than an irrational one. Furthermore, his language-based theory of knowledge would be similar to Franz Boas, Edward Sapir, and Benjamin Lee Whorf’s linguistic relativity hypothesis in cultural anthropology, since language should underlie a people’s thought (or collective worldview). Durkheim’s following statement appears to suggest this affinity:

[L]anguage is not a sort of external garment that would clothe thought from the outside without ever being able to satisfactorily fit. In reality, language is rather an integral element of thought. Language makes possible thought at least as much as it presupposes it. Without language, thought could not go up very high; for all the somewhat complex forms of mental life could not have been constituted without the help of words. Hence, with words, with language, we have something of thought and, consequently, to study language is, if one knows how to do it, to study thought itself. (Durkheim [1938] 2014, p. 70 = [1977] 2006, p. 57, see, e.g., also Durkheim [1938] 2014, p. 242 = [1977] 2006, p. 209)

*The Elementary Forms of the Religious Life*, in which Durkheim deals with the comparative-mythological studies of Friedrich Max Müller, a German mythologist who conceived of ideas as impossible without language, expresses the same view of language as above (Durkheim 1912, pp. 106–107, 115 = [1915] 1964, p. 75, 81). Moreover, Durkheim’s linguistic view is apparently similar to that of Albert Schäffle: In a review of Schäffle’s *Bau und Leben des sozialen Körpers*, Durkheim mentions that this German organicist considers a people’s language to determine their way of thinking (Durkheim 1885, p. 91); the metaphor of “garment (*vêtement*)” for language also appears. Neither should Durkheim’s reference to Rousseau’s linguistic view be ignored (see Durkheim [1918] 1966 = 1960).

However, instead of following such a genealogy, I point out that Durkheim obscurely characterizes the five social facts other than language as systems of ideals: “The principal social phenomena, that is, religion, morality, law, economy, and aesthetics, are none other than systems of values and hence of ideals” (Durkheim [1911] 1951a, pp. 140–141 = 1965, p. 96). No reference to language here is more than coincidence; elsewhere, he omits language from examples of “variables” for sociology to explain, although he does include religious beliefs, moral rules, legal precepts, aesthetic techniques, and economic institutions (Durkheim 1912, p. 4 = [1915] 1964, p. 3). Therefore, it would be more natural to think that Durkheim deliberately avoided dealing with language as an ideal system. Yet, this actually suggests what role he supposed for language: It distinguishes, classifies, and transmits ideal systems such as those five social facts consisting of abstract concepts. Language is a constant that determines variables (i.e., other social facts). It is hence a social thing in the highest degree.

It is significant here to recall the explanatory principle in Durkheim’s sociology: the social division of labor. Industrialized society requires many abstract ideas for each functionally differentiated system, such as religion, law, economy, and aesthetics, and people must also use a legal code, a monetary system, and the like. Thus, language practically has a fundamental role in modern life.

However, the more important social function of language may be to constitute modern moral order: Universal human ideals like individual freedom, against whose violation modern society imposes some kind of sanction, are shared only through language, and people thereby find real binding force in such a metaphysical ideal. Stated in the Saussurean way, conceptual life is bound by the signifier. Durkheim himself insisted that a language spoken by a group of people marks their system of ideas (see Durkheim 1912, pp. 106–107 = [1915] 1964, pp. 75–76). He details:

A value judgment expresses the relation of a thing with an ideal. Yet, the ideal is given like the thing, although of a different manner; it is […] a reality in its own way. Therefore, the expressed relation unites two given terms, just like in judgment of existence. Will one tell you that the judgments of value bring ideals into play? It is, however, no different for judgments of reality, because concepts are equally constructions of the mind, and accordingly ideals; and it would not be difficult to demonstrate that they [concepts] are even collective ideals, since they can be formed only in and through language, which, at the highest level, is a collective thing. (Durkheim [1911] 1951a, p. 139 = 1965, p. 95)

However, this view of Durkheim’s contains an implicit assumption: For collective ideals, people must share the same language. In other words, the common language has to be a “non-contractual element in contract” in society, because it enables individuals to make civil or social contracts. Practically, too, the sharing of a common language would be necessary for people, despite their diversity, to contract the exchange of expertise in the social division of labor. Next, I will explore this point in terms of Durkheim’s notion of morality and solidarity.

## Language of *Gesellschaft*

### Language as medium for society’s soul

Durkheim’s view of language is closely related to his concept of *organic solidarity*. He originally intended to sociologically inquire into how society can be well-integrated despite the individual and functional differentiation in the social division of labor. He paradoxically answered that the division of labor generates a new morality for integration: Due to increased individual variation, people’s attributes are necessarily summarized in abstract humanity beyond concrete differences, and thereby the individual’s dignity is evaluated as supreme (see Durkheim [1898] 1987b, pp. 271–272 = 1973, pp. 51–52, Durkheim 1897, p. 378 = [1951] 1966, pp. 333–334).

The “cult of the human individual” that Durkheim calls *moral individualism* originates with the progress of the social division of labor (see Durkheim [1906] 1951c, p. 84 = 1965, p. 59). He distinguished this moral type of individualism from the egoistic type, like Herbert Spencer’s utilitarianism, which separates people, impelling them to focus on their own interest, and disrupts solidarity (Durkheim [1898] 1987b, pp. 262–263 = 1973, pp. 44–45, Durkheim 1897, pp. 382–383 = [1951] 1966, pp. 336–337). He consistently referred to moral individualism as the basis for integrating French society:

[T]here exists another individualism [distinguished from egoistic individualism] that is less easy to beat. […] [T]his is the individualism of Kant and Rousseau, of the spiritualists, that is, the one which the Declaration of Human Rights attempted, more or less successfully, to formulate and which is currently taught in our [France’s] schools and has become the foundation of our moral catechism. […] Far from making personal interest the objective of conduct, it [moral individualism] sees the very source of evil in all which is personal motive. (Durkheim [1898] 1987b, p. 263 = 1973b, pp. 44–45)

The morality of respect for humanity is a rational laic morality (see Durkheim [1925] 2012, p. 33 = [1961] 1973, pp. 11–12) and the only possible religion in modernity. The human being has become a god for human beings themselves (see Durkheim 1897, p. 378 = [1951] 1966 pp. 333–334). This “religion of humanity” (Durkheim [1898] 1987b, p. 271 = 1973b, p. 51) is none other than *civil religion* in Robert Bellah’s idiom^3^: Collective life in modern society remains underpinned by a religious belief equivalent to the ideal of society itself. As has been shown, Durkheim himself espoused the ideals of freedom and equality embodied in the French Revolution and its inheritor, the Third Republic of France, in which he lived (see Tiryakian 1978). Despite having been born a Jewish rabbi’s son, he was a “true child of the Enlightenment” (Hughes 1958, p. 280) and the “highest priest and theologian of the civil religion of the Third Republic” (Bellah 1973, p. x).^4^

In this regard, Durkheim was a modern liberal individualist, contrary to the usual understanding of him as a collectivist (see also Fournier 2007, p. 16 = 2013, p. 7). He originally chose collectivism to advocate for individualism. In his opinion, beliefs (i.e., conceptual thoughts or ideals) are only active when they are shared (Durkheim 1912, p. 607 = [1915] 1964, p. 425). Society, he remarks, “can last only on the condition that there exist sufficient similarities between all its members, that is to say, on the condition that they all reproduce, in differing degree, the essential characteristics of the same ideal which is the collective ideal” (Durkheim [1925] 2012, p. 97 = [1961] 1973, pp. 87–88). Moral individualism must accordingly be collective to be real; in this sense, society realizes modern free individuals. “[F]reedom became a reality only in and through society” (Durkheim [1906] 1951c, p. 79 = 1965, p. 55). Society and the individual are not always antagonists. Rather, society underlies the religion of humanity that liberates individuals from an old order. “Collective life is not born from individual life, but it is, on the contrary, the second which is born from the first. […] It [personal individuality] has nothing anti-social about it because it is a product of society” (Durkheim [1893] 1973a, p. 264 = [1933] 1960, pp. 279–280).

Durkheim’s linguistic view, too, fits this thought. To clarify, the comparison of his social theory with Ferdinand Tönnies’s might be useful. This German sociologist famously distinguished between *community* (*Gemeinschaft*) and *society* (*Gesellschaft*), arguing that the former is superior to the latter in terms of morality and solidarity: Community is a living organism and genuine living-together, while society is merely a mechanical artifact and an apparent living-together (Tönnies [1887] 1922, p. 5). Here, *community* implicitly refers to a homogeneous, pre-modern collective, contrasted with a heterogeneous modern society, and Tönnies considered language to be crucial in community-building (communitization; *Vergemeinschaftung*) as a natural foundation for solidarity:

The true organ of understanding (*Verständnis*)^5^ […] is language itself […]. Language has […] neither been invented nor […] arranged as a means or tool by which one makes oneself understood. It is itself a living understanding, and simultaneously [that understanding’s] content and form. As is the case with all other conscious expression activities, *linguistic* expression is the spontaneous outcome of deep feelings and prevailing thoughts, and it does not work, as an artificial tool with no natural underlying understanding, for the *intention* for one to make oneself understood. (Tönnies [1887] 1922, p. 20, emphasis in original).

In Tönnies’s view, the unity of community is a given due to common language, which underlies people’s mutual understanding and trust, although morality is lost in the transition from organic community to mechanical society—an aggregate of utilitarian individuals (see also Tönnies [1887] 1922, pp. 51–53, 238–239).

Durkheim spelled out an opposite perspective to that of Tönnies’s critical view of society: “[T]he point where I separate myself from him [Tönnies]—it is his theory of *Gesellschaft*” (Durkheim 1889, p. 421 = 1972, p. 1198, emphasis in original). While agreeing to the thesis of transition from community to society, Durkheim wrote, “the life of large social aggregations is as totally natural as that of small aggregations” (Durkheim 1889, p. 421 = 1972, p. 1198). He even insisted that society rather than community is a living organism and morally superior; while pre-modern communities remain relatively self-sufficient segments and only form a mechanical solidarity, the division of labor in modern society leads to interdependent, organic solidarity among functionally specialized parts or individuals, and this becomes the source for respect of abstract human being (see Durkheim [1893] 1973a, p. 396 = [1933] 1960, p. 401; see also Tiryakian 1978, pp. 197–199). The transition from community to society results in moral progress, not retrogression.

In this context, language matters to Durkheim for a reason opposite to German organicism. For an efficient, trusted social division of labor, a whole society must have been integrated by a common ideal, which is “[T]hat in which all the people of a same group communicate; it [the common ideal] is that which especially gives them the moral unity” (Durkheim [1906] 1951d, p. 99 = 1965, p. 68); the common ideal is, however, shared through the common language. Durkheim originally thought that religion, a system of ideas as well as a system of practices, must translate realities into an “intelligible language” (Durkheim 1912, pp. 203, 322–323, 611–613 = [1915] 1964, pp. 141, 225, 428–429). This is also true of civil religion. Society requires a common language so that its members share civil-religious ideals like liberty, equality, fraternity, and corresponding worldviews.

Thus, Durkheim’s theory of knowledge presupposes the linguistic unity of society for its moral unity. Put differently, the sharing of a language underlies civil society–building based on abstract human ideals. Suggestively, Durkheim referred to ideals that enable moral individualism as “society’s soul (*l’âme d’une société*)” (Durkheim [1925] 2012, p. 124 = [1961] 1973, p. 123; see also Tada 2018b). He certainly treated society like a living body with the German-organicist metaphor, but he found that his genuine research object—the French civil society or *Gesellschaft*—was much more labor-specialized, individualized, and accordingly less race- or ethnicity-focused than Germany (see Durkheim [1887] 1975a, p. 482). Therefore, its functionally differentiated organs had to be integrated by universal ideals since the Revolution, not by some ethnic substance called *Volksgeist* (*l’âme des peoples*) contained in the German concept of *Gemeinschaft*. With French society in mind, Durkheim remarked, “To be attached to society is to be attached to the social ideal” (Durkheim [1925] 2012, p. 93 = [1961] 1973, p. 82) and an “[I]deal type which each society asks its members to realize [is] the keystone of the whole social system and gives it the unity” (Durkheim [1906] 1951c, pp. 81–82 = 1965, p. 57).^6^ Durkheim deemed France a “community of ideas” (Cladis 1998, p. 28).

This view shows an affinity with Ernest Renan who, with the phrase “plebiscite of every day” (Renan [1882] 1887, p. 307), defined *nation* as a subjective collective beyond objective-seeming elements such as ethnicity.^7^ In fact, as Schnapper ([1994] 2003, p. 28) also notes, Durkheim implicitly identified the concept of society with French civil yet national society, whose integration has been grounded not on ethnic commonality but on civil religion—more exactly, “national civil religion” (Wallace [1977] 2000, p. 221). Because of this methodological nationalism, there was virtually no distinction between modern human ideals and national ideals for him: Moral individualism is “the only belief system that can ensure the moral unity of *this country* [France],” and “if there is a country among all others where the cause of [moral] individualism is truly national, it is our own” (Durkheim [1898] 1987b, pp. 270, 274 = 1973b, pp. 50, 54, emphasis added).

Durkheim then thought that the common language must be pre-shared for the moral unity of France so that the common ideal can be shared in national society despite its differentiation and individualization. Important here is that society could contain a linguistic diversity at least at the beginning, because it is not always the grown form of one linguistically homogeneous community. Rather, society (*Gesellschaft*) would usually come into existence through a cross-cultural linkage of communities (*Gemeinschaften*), which would respectively have their own vernacular and *Volksgeist*. Therefore, to presume the linguistic homogeneity of society to be a natural given is naïve. Durkheim’s French society achieved linguistic unity by artificial means like school education, and the French language thereby became the language of *Gesellschaft* with which society’s soul is shared beyond the languages of *Gemeinschaften*.

### Organic solidarity and public education

In Durkheim’s perspective, modern, secular human ideals are religious yet disenchanted, non-mysterious civic beliefs.^8^ His adaptation of organicism was also intended to nurture citizens; for this purpose, he even openly displayed his faith in the State (*l’État*) as the control entity of the social organism, calling it the “cerebrospinal system” controlling other organs (Durkheim [1893] 1973a, p. 198 = [1933] 1960, p. 219) or the “very organ of social thought” (Durkheim [1950] 1995, p. 87 = 2012, p. 42). For Durkheim, the modern state was the organ of rationality that evolutionarily emerged for unifying social functions originating in the social division of labor (see Badie and Birnbaum [1979] 1982, pp. 27–37). In this regard, the State (i.e., government) was the practical backing for moral individualism. Durkheim manifestly hoped that society would realize modern ideals with the assistance of state regulations:

It [moral individualism] is expressed theoretically in the Declaration of Human Rights […]; it is, however, far from deeply rooted in the country [France]. […] [F]or instituting moral individualism, it is not enough to declare it and to translate it into beautiful systems [of thought]; society must be arranged [practically through the State] in such a way as to make this constitution possible and durable. […] [I]t is the State which creates them [individual rights], organizes them, and makes them into reality. (Durkheim, [1950] 1995, pp. 95–96 = 2012, p. 51)

Durkheim may have expected an enlightening government due to his ethnic marginality as a rabbi’s son. Of course, he recognized that the State could be oppressive to the individual. The Dreyfus Affair, an anti-Semitic “collective effervescence,” would be an example; Durkheim resolutely advocated for Albert Dreyfus, the Jewish artillery officer from Alsace. However, this attitude derived from his civil loyalty to the national credo of modern France, not from ethnic solidarity with fellow Jews or from antipatriotic or anarchist feeling. Rather, Durkheim adhered to the French nation-state beyond origin, race, or religion as an embodiment of universal, modern human ideals.^9^ He had confidence in the State because he, as Bellah (1973, pp. xvi–xvii) characterizes, was not a conservative but a political liberal. Thus, he believed the State to liberate the individual from the tyranny of pre-modern collectivities (Durkheim [1950] 1995, pp. 93–99, 103–104 = 2012, pp. 49–54, 58–59). Today, his political stance would be classified as *liberal nationalism* (Adachi 2013, pp. 67–90).

In liberating individuals, Durkheim insisted on the importance of education to the moral unity of civil society. Social facts are not those which one has made alone but which one “has received through education” (Durkheim [1895] 1956, pp. 3–4 = 1982, p. 50). Durkheim’s sociology of religion attracts much attention, but his sociology of education was much more theoretically important for him since religion is underpinned by education. To begin with, he perceived traditional religions to no longer function in rebuilding favorable solidarity among modern people (Durkheim 1897, pp. 430–432, 440–441 = [1951] 1966, pp. 374–376, 383). Of course, nor does education itself have such a function. It is a mere reflection of society because of its dependence on the moral condition of society (Durkheim 1897, pp. 427–428 = [1951] 1966, pp. 372–373). However, this implies that, insofar as society is morally healthy, education can reproduce it. In this regard, Durkheim conceived school as having a comparable function with that which church once had for traditional religion. School spreads civil religion among the public and maintains it within the whole society.

Durkheim then expected the State—the cerebrospinal system—to work for schools as the Vatican does for Catholic churches: School education must be state-controlled for moral individualism to prevail among all children (see Durkheim 1922, pp. 60–61 = 1956, p. 80). While Max Weber famously characterized the nature of the modern state as a monopoly on violence, Durkheim seemingly considered it, to borrow Ernst Gellner’s ([1983] 2008, p. 33) words, to lie in the “monopoly of legitimate education.” Durkheim utters, “As long as education is an essentially social function, the State cannot be disinterested in it. On the contrary, all that pertains to education must to some degree be submitted to its influence” (Durkheim 1922, p. 60 = 1956, p. 80). Certainly, he himself carefully added that he never meant that the State should monopolize education (Durkheim 1922, pp. 60–61 = 1956, p. 80). However, he simultaneously insisted on an active intervention of the State into education and stressed that even non-public-school teachers must be qualified by the State (Durkheim 1922, pp. 61–62 = 1956, pp. 80–81), saying, “There is no school which can claim the right to give, with full freedom, an *anti-social* education” (Durkheim 1922, p. 61 = 1956, p. 80, emphasis added). State-controlled education must foster the cultivated (and, in this regard, similar) citizens that modern national society needs for its own integration (see Durkheim 1922, pp. 115–117 = 1956, pp. 120–122).

Durkheim’s argument about education had the goal of building French civil society: “[E]ducation must assure, among the citizens, a sufficient community of ideas and sentiments without which any society is impossible” (Durkheim 1922, p. 60 = 1956, p. 80). He then conceptualized civil society–building as led by the State. Note that around that time many non-public schools remained, almost all of which were Catholic schools (see Tanigawa 2015, chap. 6–7). In this situation, Durkheim believed that children, bearers of the future of France, must be first taught not pre-modern religious morality but modern civic morality through “laic education (*éducation laïque*)” (Durkheim [1925] 2012, p. 237 = [1961] 1973, p. 260). He sought the rationalization and laicization (*laïcisation*) of morality through school education (Durkheim [1925] 2012, p. 33 = [1961] 1973, pp. 11–12, Durkheim [around 1908–1910] 1992, p. 611). This educational thought precisely corresponded to the principle of *laïcité* (laicism) in the Third Republic:

[I]n spite of all the dissidences, there are at present, at the basis of our [French] civilization, a certain number of principles that, implicitly or explicitly, are common to all, that very few, in any case, dare to deny overtly and confrontationally: respect for reason, for science, for ideas and sentiments that are at the base of democratic morality. The role of the State is to bring out these essential principles, to have them taught in its schools, to ensure that nowhere is children left ignorant of them, that everywhere they would be spoken of with the respect which is due to them. (Durkheim 1922, p. 62 = 1956, p. 81)

Historically, a republican educational system for nurturing citizens was an ardent wish of the revolutionaries. Despite several plans, the First Republic (and also the First Empire of Napoleon) had virtually given up modernizing (laicizing) primary education while nonetheless creating a prototype for an elite-education system: the secular system of secondary and higher education like École Centrale (or Lycée), Grande École, and Université Impériale (Tanigawa 2015, pp. 105–124). Eventually, in 1833 during the anti-Catholic July Monarchy, the laicization of primary education was undertaken with the so-called Guizot Law, which declared that each municipal commune with over 500 population must have one public primary boy’s school and each prefecture one normal boy’s school (Tanigawa 2015, pp. 151–152; Baubérot [2000] 2008, chap. 2–3). With ensuing obstacles and detours such as the Falloux Law in 1850 during the Second Republic or the intensified conflict between the so-called “two Frances” in the Second Empire, the 1881–1882 Ferry Laws in the Third Republic introduced the three principles of “obligatory, free of charge, and laic” into (public) primary education. Then, the Goblet Law in 1886 stipulated the laicity of public school teachers.

Thus, since the 1880s, the Third Republic had excluded members of religious orders from education administration, religious instruction from the school curriculum, and members of religious orders from teaching in public education. The law in 1904 forbade monks and nuns to teach even at private schools. Laicization then culminated with Prime Minister Émile Combes’s draft law for the separation of church and state, which was enacted with a slight change by Maurice Rouvier’s cabinet in 1905 (Tanigawa 2015, pp. 212–237). The law confined religion to the private sphere, while primary school teachers were expected to work as “priests of the Republic” and acted as such with missionary fervor (Tanigawa 2015, pp. 200–219).^10^

Durkheim thought that civil religion should fill the moral emptiness originating in laicization, and he gave the State a crucial role: “[T]he role of the State in this respect is what it was formerly. It rests with it [the State], so to speak, to organize the cult, to preside there, and to ensure its regular functioning and development” (Durkheim [1950] 1995, p. 104 = 2012, p. 59). Thus, Durkheim regarded the state-controlled school as a kind of church of civil religion. He also insisted that no school is at liberty to educate in a way that denies the free society of modern France or harms its solidarity, even saying that public schools (*écoles publiques*) “are and must be the guardians par excellence of our [French] national type” (Durkheim [1925] 2012, p. 27 = [1961] 1973, pp. 3–4). In Durkheim’s view, state-controlled education incorporates moral individualism into French national identity, and thereby the French national society becomes a civil-social reality *sui generis*, which would impose sanctions upon behaviors that compromise sacred human ideals such as freedom and equality: “[T]here is one [society] that enjoys a genuine primacy over all the others. It is the political society, the fatherland (*patrie*) […]. [T]he school is the only moral environment where the child could methodically learn to know and love it [the fatherland]” (Durkheim [1925] 2012, p. 90 = [1961] 1973, p. 79; see also Tiryakian 1978, p. 197).

However, it was Durkheim’s linguistic sociology that underlay his educational sociology, which in turn underpinned his civil-religious ideal. As seen above, conceptual thinking requires language. Suggestively, Durkheim believed that unorganized thought reaches maximum intensity in children (Durkheim [1938] 2014, p. 394 = [1977] 2006, pp. 343–344). Although he considered conceptual thought itself to be universal and transcending cultures, Durkheim presupposed a hierarchy between children’s and adults’ thinking abilities: Children think less abstractly than adults and are animal-like. In this sense, “[A] human being is only a human being to the degree that the person is civilized” (Durkheim [1906] 1951c, p. 79 = 1965, p. 55). This difference would, along with Durkheim’s theory, derive from the fact that children are neither well-learned nor well-socialized by language: Language brings a logical, reflexive, and self-controlled state to thought—it pulls thought out of its natural state (Durkheim [1938] 2014, p. 394 = [1977] 2006, p. 344). He went on to say that

[i]t is words that introduced distinctions into the framework of our representations. For the word is discrete; it has a definite individuality, that is, clearly defined limits. […] Of course, it would be completely wrong to say that language must do everything, that it is the sole agent of distinctiveness and clarity. Nothing can exempt consciousness from the effort of grasping a confused group of thoughts, of isolating it, of concentrating upon it all the light which is within consciousness, and of illuminating it in such a way as to highlight the unnoticed parts of which consciousness is composed. It is this attention and concentration which are the active tools of all intellectual analysis. However, the products of this analysis would remain remarkably precarious, they would soon evaporate, and thought would return to its initial confusion, if the word did not fix the products, not give them an existence, an individuality, a consistency which permits them to survive. (Durkheim [1938] 2014, pp. 394–395 = [1977] 2006, p. 344)

This view is perhaps easier to understand through comparison with the philosopher Henri Bergson, an anti-rationalist French philosopher and contemporary of Durkheim. Criticizing the modern-scientific understanding of time, Bergson insisted that human freedom lies in the pre-linguistic sphere called *pure duration* and said that the “homogeneous time [assumed in modern science] is […] *an idol of language*, a fiction whose origin we easily find” (Bergson 1896, p. 231, emphasis added). By contrast, Durkheim believed that people’s sharing of modern human ideals embodies individual freedom, ultimately grounded on (shared) language:

Language is a product of society, which, like morality, expresses one of the physiognomies of society. To learn words is not only to learn sounds but also to learn ideas. A dictionary contains a whole way of thinking. In a language, there is a mentality of its own. By learning a language, we store up a whole system of ideas that express reality and a whole set of ways of seeing things. It is by learning the mother tongue that our mind is formed. Language comes to us from social education. (Durkheim [around 1908–1910] 1992, p. 619; see also Durkheim [1925] 2012, p. 83 = [1961] 1973, p. 69)

Thus, Durkheim justified the exclusive use of French for instilling “society’s soul” into children at State-laicized schools. I shall argue about this point next. According to Durkheim’s (and the Third Republic’s) logic, the moral unity of society requires monolingualism, in which one language is universally shared as the ultimate social thing.

## National integration and language of freedom

### French monolingualism for the hastened nation

Durkheim conceived that state-supervised schools function as modern public facilities for socialization in a way that differs from socialization by the family as well as church: In his view, families do not encourage children to cultivate their own individualities and to grow to respect the dignity of others (i.e., to learn moral discipline for modern life). For children to adopt this universal morality, they should be released from family and interact with various people in school (Durkheim [1925] 2012, pp. 141–151 = [1961] 1973, pp. 144–157).

Durkheim thought children should not be socialized through a particularistic moral ideal because general social life was increasingly important with the development and centralization of society (see Durkheim [1925] 2012, pp. 86–87, 144–145, 220–221 = [1961] 1973, pp. 74–75, 148–149, 238–239). It is noteworthy that Durkheim referred only to French as the official language for school education, aside from dead languages like Latin and ancient Greek as secondary languages in secondary education. However, his conviction about the sole use of French at school was much less natural than might be expected today, since French was not always a lifeworldly mother tongue for children in France. France in those days was undoubtedly a multilingual state with many regional languages (patois) like Breton, Germanic languages (Alsatian, Franconian, and Flemish), Basque, Occitan, Catalan, Corsican, and Oïl (Gallo and Picard), some of which, linguistically speaking, differ considerably from French.^11^ Such languages of *Gemeinschaften*, communal rather than social facts, could not have been ignored.

Nevertheless, Durkheim gave no sociological account to multilingual France, strangely for a pioneer of the sociology of language. He would have known local speech: His hometown, Épinal in Lorraine, was near Alsace,^12^ and his first assignment as a university lecturer was in Bordeaux, in the Occitan-speaking area. It is therefore unlikely that he overlooked the country’s linguistic diversity. He may have deliberately avoided mentioning regional languages. Although indirectly, he seems to have disapproved of regional languages, still spoken at homes and in local communities, being used at school. As suggested above, he justified the exclusive use of French at school, as if it were common sense, using the expression “*our* language” (notably in singular form, and not the plural “our language*s*”) and stating that (only) French is suitable for expressing the general ideals of the French Revolution:

[O]ne could have said that we [French people] thought for humanity. […] Hence the declarations of rights valid for all the human race, for which we have been reproached so much in the name of the so-called historical method [as in Germany]. There is the way of seeing quite fundamentally inherent in our character which *our language* [French] itself bears its imprint. Because it [French] is essentially analytical, it is marvelously suited for expressing such a way of thinking. (Durkheim [1925] 2012, pp. 252–253 = [1961] 1973, p. 279, emphasis added)

Since Durkheim gave no concrete evidence for the essential analyticity of French, his insistence resembles ideological myth, given Antoine de Rivarol’s well-known proverb, “What is not clear is not French,” from his 1783 *Discourse on the Universality of the French Language* (*Discours sur l’universalité de la langue française*). Durkheim, despite his language-relativist views, seems to have adopted the hierarchy of analytical quality among languages. This belief in the supremacy of French over other languages is already a kind of linguistic nationalism, but it is not limited to Durkheim. Combined with the loyalty to the State, such a belief was seen in the French liberal intelligentsia of those days, as Hughes points out, “[T]he French intellectuals were not even remotely tempted to renounce their national allegiance. […] The French writer or scholar simply assumed that his country was the center of the civilized world, as its language was the most perfect vehicle for intellectual communication” (Hughes 1958, p. 59). However, what is more noteworthy here is that the faith in the supremacy of French could be directed against not only foreign languages but also regional languages inside borders: patois are less clear than French, as Hamel, a primary school teacher in Alsace, may have believed. Interestingly, Durkheim remarked that two dead languages, Latin and ancient Greek, would be helpful for children to learn abstraction and analysis (Durkheim [1938] 2014, pp. 310–317, 395–396 = [1977] 2006, pp. 270–277, 345). On this score, Durkheim acknowledged room for linguistic compromise with the church. In contrast, he totally ignored the possibility of using or teaching the living languages of France other than French in schools, although language confined to the private sphere would have little chance of survival.

Originally, French monolingualism was found among republicans rather than conservatives. Republicans thought that multilingualism could destabilize the national unity based on common ideals. Unsurprisingly, those who spoke French “correctly” at the time of the French Revolution constituted only 12–13% of the population, although the language was used to write the Declaration of Human and Citizens’ Rights (*Déclaration des Droits de l’Homme et du Citoyen*) in 1789 (Hobsbawm 1992, p. 60). Certainly, French had already replaced Latin as the language for official documents since the Ordinance of Villers-Cotterêts in 1539. The aristocracy had also been Francicized, but it still belonged to the pre-national history of France.

Nonetheless (or therefore), the revolutionary government insisted on linguistic uniformity as a condition for full French citizenship and nationality (Hobsbawm 1992, p. 21). Regional languages were perceived as obscurant and counterrevolutionary obstacles to realizing the declaration in the 1793 Constitution that “The French Republic is one and indivisible.” For instance, Bertrand Barère claimed in his 1794 report:

We revolutionized the government, the laws, the customs, the lifestyles, the costumes, the commerce, and the thought itself; thus, we shall also revolutionize the language that is their daily instrument. […] Federalism and superstition speak Low Breton; immigration and hate of the Republic speak German; counter-revolution speaks Italian, and fanaticism speaks Basque. We shall break these instruments of misfortune and fallacy. (Barère 1794, p. 8)

Four months after this, Henri Grégoire, who had known through his cross-country survey in 1790 that the native French speaker was a minority, also declared:

[W]e can make uniform the language of a great nation so that all the citizens who compose it can communicate their thoughts to each other without obstacles. This undertaking, which was not fully carried out among any people, is worthy of the French people, who centralize all branches of social organization, and who must be intent on devoting rather to, in a single and indivisible Republic, the sole and invariable use of the language of freedom (*langue de la liberté*). (Grégoire [1794] 1974, pp. 200–201)

This speech was titled “Report on the Necessity and Means to Annihilate the Patois and Universalize the Use of the French Language.” Revolutionists dismissed the freedom of language for the sake of French, the language of freedom, despite acknowledging the freedom of religion. “[T]he language of a free people must be one and the same for all” (Barère 1794, p. 11). The “one and indivisible” linguistic unity was considered indispensable to French unity. The Third Republic radically enacted this assimilationist language policy. Schnapper describes:

Indeed, the primary school teachers (*instituteurs*) of the Third Republic nationalized the children of the peasants in the provinces of France and those of immigrants, teaching them French and calculation, if necessary, brutally, and forbidding them to use the language of their parents. (Schnapper [1994] 2003, p. 97).

Spreading the French language was teachers’ most important mission for consolidating the indivisibility of the Republic, which was to consist of enlightened citizens. In 1863, about 11% of schoolchildren (ages seven to thirteen) spoke no French, and another 37% spoke or understood it but could not write it (E. Weber 1976, pp. 67, 310–314) but, in the 1880s, the Third Republic began to promote the sole use of French language at school (i.e., using the so-called direct method) (Hara 1990, pp. 215–218). Just as Luther translated the Bible into German so that believers could know the doctrine themselves, the laic Republic believed that civil religion, or laic morality, had to be delivered directly to all citizens through the universal yet national language, overcoming linguistic barriers not only of Latin but also of patois; under the hegemony of the only official language, free and equal French citizens could join a public debate as well.

James Crawford notes, referring to monolingualization in France, “Only with the rise of modern states came the means and motivation to popularize a standard language. Languages do not mold nations, but vice versa” (Crawford 1992, p. 239). In fact, drastic nation-building from “Peasants into Frenchmen” (E. Weber 1976) in the Third Republic began with defeat in the Franco-Prussian War. As mentioned before, the defeat made it urgent to establish a strong French identity. In this light, the French nation was, in contrast to the “belated” German nation, a “hastened nation” (Watanabe 1997, p. 2) created through radical assimilationism from above, whose main means was school education. “The School had to transform into citizens the members of restricted and particular communities so as to make them participate in the universality of national citizenship” (Schnapper 2000, p. 155). Thus, multilingualism would have seemed to republicans as *linguistic anomie*, which might prevent the sharing of ideals in one indivisible nation and thereby lead to civil-moral anomie.

Finally, a situation in which the whole French nation spoke French appeared only after World War I, which broke out when the nation, not the individual, was becoming the supreme value.^13^ Interestingly, when Durkheim referred to language as a social fact in 1895, he uttered, as if self-evident, that “I am not obligated to speak *French* with my compatriots, nor to use legal currencies, but it is impossible for me to do differently. If I tried to escape from this necessity, my attempt would fail miserably” (Durkheim [1895] 1956, p. 5 = 1982, p. 51, emphasis added). However, it is doubtful that such a monolingualism had been fully realized in France. Even though French monolingualism had become a binding social fact at that time, it could properly be called a *national-social fact* underpinned by state-controlled education. Daniel Chernilo points out that Durkheim “tries to combine *sociological* and *normative* arguments” and his social theory (probably unintentionally) shows “the normative ambiguity of the nation-state’s position in modernity” (Chernilo 2007, p. 63, emphasis in original). Durkheim’s statement on French monolingualism cited above seems to be a typical example. The Third Republic’s national-educational goal was that all the people become French speakers, a goal that was to be achieved through education at any cost (e.g., loss of regional languages)—an ambition that was shared by Durkheim.

### Centralization and civil-linguistic nationalism

Historically, at the end of the nineteenth century and during Durkheim’s life, the term *regionalism* began to be used in France to refer to the movement for restoration of regions or provinces and became widespread through the activity of the French Regionalist Federation (*Fédération régionaliste franҫaise*) organized in 1900 (Endō 1992, p. 3). It was natural that such a “reactionary” phenomenon, although containing an aspect of a proxy war between state and church, appeared with the increase of unequal development between the central and peripheral areas under capitalism or during the *Belle Époque*. What is suggestive for our discussion is that the regionalisms struggled to have their languages taught at school in protest against the centralization of language (Calvet [1974] 2002, p. 239): Ferry’s school policy “condemns local languages to death” (Calvet [1974] 2002, p. 227).^14^

Nevertheless, Durkheim, a pioneer of linguistic sociology, conceived of the return to old particularism(s) to be harmful to national unity (see Durkheim 1922, p. 116 = 1956, pp. 121–122), coldly stating the following:

Today neither the commune, the department nor the province has enough ascendancy over us to be able to exert this [moral] influence; we see in them only conventional labels without any meaning. Of course, all things being equal, people generally prefer to live in the places where they were born and have been reared. But *local homelands* (*patries locales*) *no longer exist nor can they exist. The general life of the country* [France]*, definitively unified, is impervious to all dispersion of this sort*. We may regret a thing which no longer exists, but those regrets are in vain. It is impossible to artificially resuscitate a particularist spirit which no longer has any foundation. (Durkheim 1897, p. 449 = [1951], emphasis added)

Regionalism’s rise could surely be a sign that centralization, which had been advancing in France since pre-modernity and accelerated with the increase in modern mobility, had been overwhelming local communities. Regional identities (or regional-language communities) themselves might have been romantically “imagined” in competition to Paris. However, Durkheim’s sarcastic statement above would then be exaggerative and as such reveal his sense of rivalry with regionalism. He positively evaluated centralization linked with the Revolution, saying that it developed in parallel with civilization (Durkheim [1899] 1975c, p. 170, see also Durkheim [1898] 1987b, p. 266 = 1973, p. 47). According to him, centralization achieved the *moral leveling* of French national society by reducing local diversities (see Durkheim 1897, pp. 446–447 = [1951] 1966, Durkheim [1893]1973a, pp. 163–164 = [1933] 1960, p. 187).^15^ Durkheim conceived that the abolition of former provinces through the Revolution also belonged to this civilizing, moral-integration policy (Durkheim 1897, p. 447 = [1951] 1966 pp. 388–389).

Based on such “French ideology” (É. Balibar 1988a, p. 37) forcing assimilation into universal French civilization, Durkheim flatly rejected that state-controlled moralities should be mediated to individuals through decentralization (regional devolution) (Durkheim 1897, pp. 448–449 = [1951] 1966, pp. 389–390). He clearly contrasted, for instance, Alexis de Tocqueville, who, praising local autonomy, said in his *Democracy in America* that “municipal corporations (*institutions communales*) are to liberty what primary schools are to knowledge” (de Tocqueville 1835 vol. 1, p. 83; see also pp. 153–155). Durkheim never acknowledged such federalism. Rather, to revitalize local communities could produce an effect opposite to his purpose of overcoming the “poverty of morality” (Durkheim 1897, p. 445 = [1951] 1966, p. 387). As a necessary consequence in his theory of the transition of *Gemeinschaft* to *Gesellschaft*, moral particularities would (or must) be lost with growing interrelationships between local-territorial, segmental aggregations (Durkheim 1897, p. 447 = [1951] 1966, p. 388), even suggesting that this theory is true of language:

It [the segmental organization] loses more and more it[s former relief] as societies develop. Indeed, it is a general law that partial aggregates which belong to a larger aggregate see their individuality becoming less and less distinct. Along with the familial organization, local religions disappeared irreversibly; there only remain local customs. Little by little, they merge into each other and unite together at the same time that *dialects and patois come to be resolved into one and the same national language, at the same time that the regional administration loses its autonomy*. (Durkheim [1893] 1973a, pp. 163–164 = [1933] 1960, p. 187, emphasis added)

This indicates that *language* in Durkheim’s social theory meant the language shared in national civil society, that is, the language of freedom. In this regard, he leaned toward the radicalism of Jacobin republicanism (liberal left): He thought that any re-empowerment of local community leads to retrogression from modernity to pre-modernity, since decentralization would disturb the moral leveling of citizens and its underlying *linguistic leveling* and, in consequence, dissolve the organic whole of civil society into segmental communities again. Thus, in Durkheim’s view, “antisocial provincialism” (Cladis 1998, p. 29) had to be blocked by state-controlled education.

As is known, Durkheim proposed to revive occupational groups or corporations as an intermediating, secondary organ of the social organism (and, additionally, individuals can also keep a distance from the State through the secondary organ if the State were oppressive) (Durkheim 1897, pp. 435, 448 = [1951] 1966, pp. 378, 389, Durkheim [1950] 1995, p. 138 = 2012, p. 89, see also Durkheim [1893] 1973a, p. xxxiii = [1933] 1960, p. 28). Easily overlooked, this was in truth an alternative to the decentralization of power to regions (Durkheim [1893] 1973a, pp. xxxii–xxxiii, 164 = [1933] 1960, pp. 27–28, 187, Durkheim [1950] 1995, pp. 129–130, ch. 9 = 2012, p. 82, chap. 9). Hence, he even purposely named the counterplan *occupational decentralization* (*décentralisation professionelle*), which seemed to him to successfully make the social division of labor and organic solidarity compatible with each other beyond the pre-modern spatial arrangement of communities:

The only decentralization which would make it possible to manifold the centers of communal life without breaking the national unity is what could be called *occupational decentralization*. For, as each of these centers would be only the focus of a special, limited activity, they would be inseparable from one another and the individual could consequently form attachments there without becoming less solidarity with the whole. Social life can be divided, while retaining its unity, only if each of these divisions represents a function. (Durkheim 1897, p. 449 = [1951] 1966, p. 390, emphasis in original)

The linguistic integration through French was a premise for this occupational decentralization, since common language functions, in organicist manner, like nerves, which smoothly connects all functionally differentiated organs and, for civil solidarity (fraternity), transmits the same moral instructions (i.e., modern ideals) from the cerebrospinal system, the primary organ of social organism, to individuals.^16^ Therefore, local communities with their own languages were not only unsuitable as intermediating secondary organs but also had to be overcome. The same language must be, under state control, shared by the whole nation beyond regional boundaries. By doing so, French works literally as a social fact (i.e., a non-communal fact), in building the organic solidarity of the national society consisting of citizens who believe in moral individualism. To awaken among people “the faith in a common ideal” (Durkheim [1925] 2012, p. 108 = [1961] 1973, p. 102), the sharing of the language of freedom is indispensable. In other words, French became the *sacred language for civil religion*; all free and equal believers (i.e., the whole nation) must know it.

It seems that Durkheim’s linguistic view, corresponding to the education policy in the Third Republic, could be characterized as *civil-linguistic nationalism* in contrast to ethnolinguistic nationalism in then Central Europe (see also Tada 2018a, pp. 457–458). To illustrate, the distinction between civic and ethnic nations, an ideal-typical classification of nations being *jus soli* or *jus sanguinis*, is useful. Relevant to the discussion here is that the civic nation is conventionally represented by France and the ethnic nation by Germany (Brubaker 1992). Durkheim’s view of language also belongs to the former *jus soli* nation-view: A nation is a selectable, contractual community of citizens who, regardless of their ethno-communal elements, resonate with modern human ideals and speak the language of freedom that expresses those ideals. “French nationality was,” Eric Hobsbawm remarks, “French citizenship: ethnicity, history, the language or patois spoken at home, were irrelevant to the definition of ‘the nation’” (Hobsbawm 1992, p. 88). To be precise, French was the exception among languages spoken in the country. It was a symbol of the trans-communal citizenship of a revolutionary nation. In this sense, belonging to the French linguistic community was a choice, a *social* a posteriori that individual citizens could and should, in theory, acquire by will (and irrespective of their mother tongues.) As Daniel Baggioni says, “[I]n the German conception, in the beginning were language and culture, while in the French conception language is only an instrument of political and cultural, or rather ‘civilizational,’ unification” (Baggioni 1997, p. 224; see also Watanabe 1997, pp. 21–22).

Note that such a linguistic view in France is the type of linguistic nationalism that leads to linguistic imperialism or assimilationism aiming to annihilate vernaculars.^17^ Linguistic nationalism itself is quite common to modern nation-states. Hobsbawm says:

National languages are therefore almost always semi-artificial constructs and occasionally, like modern Hebrew, virtually invented. They are the opposite of what nationalist mythology supposes them to be, namely the primordial foundations of national culture and the matrices of the national mind. They are usually attempts to devise a standardized idiom out of a multiplicity of actually spoken idioms, which are thereafter downgraded to dialects, the main problem in their construction being usually, which dialect to choose as the base of the standardized and homogenized language. (Hobsbawm 1992, p. 54)

Most parts of this citation are true of France, but not in the sense of ethnolinguistic nationalism, which believes a language to ethnically demarcate a nation sharing *Volksgeist*. Contrary to such an imagined *Gemeinschaft*, modern France was not so much a reality sui generis but an imagined *Gesellschaft*, in which the universal language demarcates the universal civic nation beyond ethno-communal elements. Speaking French was synonymous with sharing society’s soul. The French nation was another kind of *Sprachnation*.^18^

Durkheim obviously presumed the “nationalist equation of one language = one nation = one state” (Auer 2005, p. 8). As Marion M. Mitchell points out, “While retaining humanity as a god, he [Durkheim] recognized the divinity of the nation” (Mitchell 1931, p. 106). But Durkheim identified the French nation with free and equal French citizens who believe in moral individualism, and therefore he transferred organicist thought from the ethnic nation (*Volksgeist*) to the civic nation (society’s soul). Then, to overcome ethno-communal differences and to form the national civil society by universal ideals, he believed that the linguistic medium for the public life had to be French. French as a social fact was ideal rather than real.

Durkheim’s linguistic nationalism was a civic type of methodological nationalism: Civil society as a *francophonie* conterminous with the national borders. According to him, “[L]anguage is fixed; it changes only very slowly, and consequently the same applies to the conceptual system that it expresses” (Durkheim 1912, p. 619 = [1915] 1964, p. 433). This can easily be reversed: People must share the same language to share a conceptual system. Since language is “the most immediate, the closest, the most easily perceptible to which the [human] mind can hold” (Durkheim [1938] 2014, p. 72 = [1977] 2006, p. 59), socialization of children is, of course, impossible without language. Civil morality can be indoctrinated and shared only through language—the same *national* language used by all citizens. In fact, for Durkheim, socialization of individuals through public education meant their nationalization (Mitchell 1931, p. 102).

As seen, Durkheim thought that language was transmitted from generation to generation. However, the transmission of regional languages was interrupted by French. French was not a social a priori that is lifeworldly given to individuals but was a social a posteriori that the free and equal nation had to acquire through a state-controlled educational system; French, then, appeared as if it were a social a priori for the community of citizens. In this sense, the language that should be transmitted from generation to generation by society was exclusively French for Durkheim, although this prestigious language of civilization and emancipation was never neutral in terms of ethnicity, regionality, nor social stratification. The linguistic assimilationism founded on the self-perceived universality and supremacy of French was itself a kind of ethnocentrism or egoism.

## Conclusion: The indivisible republic in an age of globalization

As a community of citizens, France has been virtually defined by the French language, and therefore the first-modern pressure to be linguistically homogenous remained extreme (although paradoxical) in terms of its national ideal. Non-French languages were treated in the public sphere in the way that the veil worn by some Muslim women (e.g., the burkha or the hijab) is treated today—however, language may not simply be taken off. As Durkheim believed, it is more than a mere “external garment.”

Note that French monolingualism—or, if one may, French linguistic-imperialism—is also common among French leftists (Miura 2000, pp. 114–116, 119, 128–131; Hasegawa 2000, pp. 230–231). Symbolically, Jules Ferry, who established the Republic’s laic school, made a huge contribution to expanding the French colonial empire, based on his insistence upon a civilizing mission (Miura 2000, pp. 121–123). Since the Deixonne Law in 1951 and subsequent ethnic revival in the 1960s, leftists in France have advocated for regional languages (see also Takahashi 2013/14). However, in 1999, when the Constitutional Council (*Conseil Constitutionel*) judged that ratification of the Council of Europe’s European Charter for Regional or Minority Languages was unconstitutional, Jean-Pierre Chevènement, then interior minister and a member of the socialist party, was also against ratification of the Charter, saying that the recognition of regional languages “balkanizes” the one and indivisible Republic (The sentence cited in this paper’s epigraph from *Charlie Hebdo* also appeared during the period in which this dispute over the Charter raged). Before these incidents, the sentence “The language of the Republic shall be French” had been added to Article 2 of the Constitution under the socialist party’s regime in 1992, just before the ratification of the Maastricht Treaty by referendum. The original intention of this amendment was a cultural-differentialist resistance against English imperialism (or American neo-liberalism),^19^ but such a re-Francization contained a typical French-nationalist sense of crisis in which French is losing its former universality in international society and might become a local language in the global division of labor. One could say that such resistance to English, *a global-social thing in the highest degree*, is a form of regionalism.

Consequently, such a “pro-French” movement would be linked with the ethnicization of the French language such that the line between left and right disappears (see also Miura 2001). At the very least, the amendment to the Constitution is oppressive to linguistic diversity; the reality of France is still (even more so) multilingual. Bernard Cerquiglini (2008) has reported that 75 regional or minority languages are spoken in present-day France, including immigrant communities’ languages in the mainland as well as indigenous languages in overseas departments and territories. On the whole, the nationalist illusion of linguistic homogeneity will become increasingly irreal due to full-scale globalization. National societies are facing multilingualization and/or Anglicization.

Meanwhile, in today’s France, another issue concerning national integration has emerged: French society, contrary to the national ideal, has not always allowed immigrants to fully assimilate (not: immigrants themselves have not assimilated); therefore, young people of immigrant origin—especially from a Muslim background—experience a sense of alienation from society. Such a sense is heightened more because they have share both French language and moral individualism. A symbolic and paradoxical incident happened in 1989, the bicentennial year of the Revolution: when three junior-high schoolgirls were expelled from school for refusing to remove headscarves (hijabs) in the classroom, five famous republican intellectuals proclaimed, “Teachers, let’s not give in! (*Profs, ne capitulons pas*!) ” in support of laicization in public education. In such a national-republican atmosphere, it was no wonder that some young people of immigrant origin were, although sufficiently “secularized” by the French standard, attracted to a different community, one unfortunately containing militant extremism.

The problem seems to be, rather, the fundamentalization of laicism linked with civil religion. To threaten freedom of belief by violating minorities’ dignity is acknowledged by the new *communautarisme*—this French word would denote tribalism rather than communitarianism in English—that chants “*Je suis Charlie*.”^20^ Emmanuel Todd criticizes this dogmatic, exclusionist radical laicism, saying: “This new kind of faith must, just as much as Catholicism, Protestantism, Judaism, and Islam, be held outside public schools” (Todd 2015, p. 209). This interpretation is persuasive, since an unquestioning worship, of whatever thing, could certainly be a re-enchantment or de-laicization. However, chauvinism based on laic morality is itself not a new phenomenon. Even Durkheim asserted that “[t]here could not be a question of granting to the majority the right to impose its ideas on the children of the minority” (Durkheim 1922, p. 62 = 1956, p. 81).

That Durkheim, a person of minority origin, expected laicism to lead to freedom and equality is understandable. But he was also aware that members of a religious minority group display stronger internal solidarity and identify themselves as “us” when fighting external hostility (Durkheim [1925] 2012, p. 221 = [1961] 1973, pp. 239–240). In this sense, ultra-laicism like a “tyranny of the majority,” using de Tocqueville’s (1835 vol. 2, pp. 41–42) expression, would cause a vicious circle and end up bringing the opposite of the desired effect: a divided Republic. By the standards of today’s global society, such radical laicism would only be a form of particularism or tribalism in fanatical obeyance to a particular ideal. Nor should it be overlooked that the majority’s “right to difference” in the public sphere is implicitly granted in France; to further laicize France, they must remove “ethno-French” veils such as the secular observation of Sunday (the traditional Christian day of rest) or Easter (the holiest Christian feast day) and the exclusive use of the French language in civil spheres like politics, administration, or the judiciary (see Nakano 1996, p. 77). The non-laicity of these cases should be seriously considered, since today’s rightists often use the majority’s “Frenchness” as a cultural-differentialist reason to exclude minorities from national society.

Some leftists and rightists in France are akin to each other, backed by the golden law of laicity and by their own belief in the French nation. Indeed, such a minority-suppressing coalition of two divisive ideologies can be regularly found in modern French history. Jean-Paul Sartre has written:

The anti-Semite accuses the Jews of *being* Jews; the democrat would easily reproach them for *considering* themselves to be Jews. Between their adversary and defender, the Jews seem to be in quite a bad state: It seems that they have nothing else to do but choose the sauce with which one will eat them. (Sartre 1954, p. 69, emphasis in original)

Inclusionist republicans are not always emancipators. They force minorities to become the universal individual transcending ethnicity, being ignorant of their own ethnocentrism. Against this false-universal particularism, Sartre proposed a “concrete liberalism” (Sartre 1954, pp. 177–179) that denies the abstract subject of human and civil rights as a myth, saying that “French people will not be free as long as Jews do not enjoy their rights to the full” (Sartre 1954, p. 185).

Notably, Durkheim, despite his oppressive (or cultural-hegemonic) opinion on minorities as seen above, also noticed that French cosmopolitanism could become exclusionist patriotism, and had frankly admitted that the French national character, because of its simplifying rationalism, is prone to overlook complexities that cannot be imagined in abstract form (Durkheim [1925] 2012, pp. 231–232, 253 = [1961] 1973, pp. 252–253, 279). Characteristically, he still reduced this national “simplistic mind (*esprit simpliste*)” (Durkheim [1925] 2012, pp. 236, 238 = [1961] 1973, pp. 258, 262) to the French language:

Our language itself is not made for translating the obscure underneath of things that we can feel well but not clearly understand. Precisely because our language is analytical, it expresses well only those things that are analyzed, in other words, resolved to their elements […] What it [French] seeks is the simple […]. As for the aspect that takes on the whole as a whole, as for what makes its unity, continuity and life, it [French] is in large measure uninterested. (Durkheim [1925] 2012, p. 232 = [1961] 1973, p. 253)

Durkheim possibly adopted organicist theory to overcome the limitations of simplistic French thought that seemed to him to be conditioned by the French language. Indeed, while seeking to realize the modern human ideals of the Revolution, he criticized the revolutionists for their social atomism that held all social things to come from individuals (Durkheim [1925] 2012, pp. 234–237 = [1961] 1973, pp. 256–259). By contrast, Durkheim conceived that civil society must be an organic whole or reality sui generis that is irreducible to individuals in advocating for their freedom.

Yet, Durkheim’s social organicism—we could call it *civil-social organicism*, which aims to sociologically supplement the atomistic theory of the social contract—presupposed a centralized state that attempted to eradicate local speech communities. The haunting historical lesson is that the anti-Semitic fascist regime followed the Third Republic in which Durkheim had invested so much of his faith; furthermore, it made the first official modification to the basic policy of eradicating minority languages that had been pursued since the Revolution. Responding to regionalist insistence by right-wing nationalists in the wake of the conclusion of the Armistice of June 22, 1940 with Nazi Germany, the Vichy regime allowed the teaching of dialectal languages (*langues dialectales*) as an optional subject at primary school (Miyajima 1998, p. 176; see also Miura 2000, p. 119). This ironic phenomenon—the official recognition of the linguistic right by the fascist regime—must not be simplistically dismissed as resulting from obscurantism. Rather, it seems to be a dialectic (or viciously cyclical) product of civil nationalism and ethnic nationalism in France. An uncompromising belief in the nation, whether leftist or rightist, can always invert into the principle of exclusion and consequently produce a reaction by the excluded people.

## Footnotes

1 When referring to or citing from Durkheim’s (and Fournier’s) texts in this paper, I translated from the original French into English myself, although I drew partly on existing English translations, whose publication years and corresponding page numbers I include following an equal (=) sign. Additionally, for the readers’ convenience, I added the date of the original publication in square brackets in the citations and the reference list.

2 Suggestively, Tiryakian (1978, pp. 212–213) indicates the affinity of Durkheim’s knowledge theory with ethnomethodological studies and Edmund Husserl’s concepts like *Lebenswelt*.

3 To be precise, the term “civil religion” was first used by Jean-Jacque Rousseau (1762, Livre IV, ch. 8). However, in sociology, it became well-known through Bellah (1967), who indicated a non-laicized, Judeo-Christian basis for U.S.-American civil society with this expression. By contrast, Durkheim certainly conceived of the ideal foundation of society as religion (see Durkheim 1912, pp. 598–599 = [1915] 1964, pp. 418–419), but he thought that the religion of French civil society (i.e., the collective belief of secularized French citizens) should be moral individualism rather than a non-disenchanted traditional religion like Catholicism.

4 Or, we should rather think here, not “despite” but “*because of* having been born a Jewish rabbi’s son.” Hughes argues that “The Republic […] was not only acceptable [to French intellectuals]. On occasion it even seemed worth defending with some vehemence. And this was particularly true of the Jews, to whom the word ‘equality’ in the national motto meant more than an empty symbol. For them republican patriotism was a natural product of profound gratitude” (Hughes 1958, p. 58). I will return to this later when referring to the so-called Dreyfus Affair.

5 As an English word corresponding to *Verständnis*, Tönnies suggests “consensus” in brackets in the portion of his original text that immediately precedes this cited portion. However, *Verständnis* does not always imply that people agree with each other. Hence, “understanding” seems to be more adequate here, because of its neutrality.

6 As for the French-German parallelism between society’s soul and *Volksgeist*, Durkheim ([1888] 1987a: 101–102) suggestively notes that *psychologie sociale* (*social* psychology), which studies (French society’s) collective ideals and sentiments, corresponds to *Völkerpsychologie* (*ethno*psychology) in Germany, which was primarily developed by Wilhelm Wundt at Leipzig University.

7 In 1882, after the 1871 defeat in the Franco-Prussian War, Renan famously asserted in his lecture that people become a nation on the foundation of their will to live together, regardless of differences of race, *language*, religion, or geography (Renan [1882] 1887: 309). He intended to justify the belonging of the ceded Alsace-Lorraine to France, acknowledging a “cultural jigsaw” (E. Weber 1976: 112) in France.

8 On this, see also Mathiez (1904), who has described the essentially religious character of the French Revolution by using Durkheim’s concept of religion. Durkheim himself shows a similar comprehension of the Revolution (Durkheim 1912, pp. 305–306 = [1915] 1964, p. 214). In Durkheim’s view, a secular phenomenon without mysterious content can also remain religious if it has a certain kind of form: dogma, symbols, altars, and feasts. For a brief analysis of the religiosity of secular (but nationalist-belief-related) objects, see also Durkheim (1899, pp. 19–23 = 2011, pp. 90–93).

9 As suggested in a footnote above, Durkheim’s devotion to the Third Republic’s civil religion is completely consistent with his Jewish origin, since modern liberalism based on the Enlightenment (at least theoretically) resulted in emancipating members of minorities as equal individuals free from discrimination by a dominant majority group, in this case, Catholic France (and, more generally speaking, modern liberalism emancipated all the people from their own conventional religious or local communities). In this regard, Durkheim’s experience in childhood of seeing anti-Semitic sentiment spread rapidly after the Franco-Prussian War could have an influence on his social theory. He notes, “It had already been seen in the eastern regions during the war of 1870; being myself of Jewish origin, I was then able to observe it closely. It was the Jews who were blamed for defeats. Finally, an outbreak [of persecution] of the same kind [as] in 1848, but much more violent, occurred in Alsace” (Durkheim [1899] 1975d, p. 252 = 2008, p. 322). In fact, the equality of Jews in the French nation remained only superficial at that time, and their “strangerness” in their fatherland (or, more concretely, their hometowns) would have been evident even in the eyes of a child. For details of the anti-Semitism and experiences of Durkheim’s childhood, see Natsukari (1978, 1996), Nakajima (1982), and Fournier (2007, partie 1, ch. 1 = 2013, part 1, chap. 1).

10 It would be worth noting that such a “mission” was not only true of primary school teachers (*instituteurs*). Fournier (2007, pp. 15–16 = 2013, p. 6) adequately points out that the Third Republic was in a broader sense the “teachers’ Republic” (*République des professeurs*), which should necessarily include Durkheim, a university teacher. Fournier says, “Émile Durkheim commits himself [to] and multiplies public interventions. In short, he is an intellectual in the spirit of the Third Republic, this Republic of teachers, whose *credo* revolves around three words: democracy, secularism and science (*la démocratie, le sécularisme et la science*)” (Fournier 2007, p. 918 = 2013, p. 730, emphasis in original). Accordingly, Bellah’s above-cited characterization of Durkheim as the “*highest* priest and *theologian* of the civil religion of the Third Republic” should be taken literally: Durkheim, as a university teacher of sociology, aimed to provide a theoretical justification for the Republic’s civil religion and thereby to support its ideals to be realized through the education system. Steven Lukes remarks, “Durkheim believed that the relation of the science of sociology to education was that of theory to practice; and, in this respect, it would become a rational substitute for traditional religion. Teachers should be imbued with the ‘sociological point of view’ […].” (Lukes [1973] 1975, p. 359). In fact, in 1902, Durkheim was assigned to the Science of Education at the Sorbonne as the replacement of Ferdinand Buisson, who, as Director of Primary Education at the Ministry of Public Instruction from 1879 to 1896, took a role responsible for implementing many educational reforms of the Third Republic’s primary sector, including the Ferry Laws (Lukes [1973] 1975, p. 360, see also pp. 375–376; Fournier 2007, pp. 501–511 = 2013, pp. 397–405).

11 The examples of regional languages come from Giordan (1984).

12 Incidentally, Durkheim’s parents came from Alsace. Yet (or therefore), it seems that they consciously reared Durkheim as “French-French” in the Latin-cultural milieu of Épinal, probably speaking (accented) French. See Fournier (2007, p. 28 = 2013, p. 18).

13 Along with the establishment of public education and the increased mobility and social exchange in industrialization and expanded traffic networks, military service contributed to the spread of French as a lingua franca. See E. Weber (1976, pp. 78–79, 84).

14 Hara (1990, chap. 2) demonstrates that it was in the 1880s when the linguistic shift from local tongues like Breton to French occurred in local print culture. This shift, as Chanet (2013) also describes, may partly have come from the regional inhabitants’ own effort to attempt to acquire French. However, in my opinion, such “voluntarism” of the populace should not be overestimated insofar as it was socially compulsory for them to achieve a rise in social status. In any case, a fact that such voluntarism suggests is that “small fatherlands (*petites patries*)” with their own languages were then enduring as autonomous communities. Linguistically speaking, *diglossia* was a social reality for many local people in France.

15 Durkheim also suggests that the driving force of the moral levelling was northern France, the center of French civilization, and that the South will totally assimilate into the North despite the former’s resistance (Durkheim 1897, pp. 67–68 = [1951] 1966, pp. 92–93).

16 Durkheim himself may not necessarily liken language to nerves, but this metaphor also appears in Durkheim’s book review on Schäffle’s *Bau und Leben des sozialen Körpers*. According to it, Schäffle thought that symbolic signs including language function as nerves, through which individuals or groups mutually exchange ideas (Durkheim 1885, p. 91).

17 In fact, a linguistic community is generally rather variable and has, at least formally, an admission-withdrawal openness in contrast to, for example, racial community. As for this, see also É. Balibar (1988b, pp. 134–136). Of course, such a flexibility of linguistic community (or language shift) can be easily associated with linguistic imperialism (Tanaka 2000).

18 It might be noteworthy that, as shown typically in the epigraph cited from Renan in the beginning of this paper, French scholars of those days seem to have tended to overlook the fact that the French nation was the sort of *Sprachnation* which was built sometimes “by means of coercion.” Those who were influenced by Durkheim’s linguistic view are not exceptions. For example, Meillet assumed monolingualism in a country as if it were a natural, given condition. He writes that except for “historical accident, the borders of diverse languages tend to coincide with those of social groups which one calls nations: The absence of a unity of language is the sign of a recent nation like in Belgium or an artificially constituted one such as in [Habsburg] Austria; language is thus eminently a social fact” (Meillet 1906, p. 1). In contrast to this fallacy of Meillet’s, which was obviously based on methodological nationalism, Mauss wisely points out in an uncompleted manuscript that nationalities (*nationalités*) create their own languages, because the language creates the nation (Mauss [2013] 2018, p. 105). This observation by Mauss can be said to have anticipated Benedict Anderson’s famous conception of the nation-state as an “imagined community.” However, Mauss primarily discusses nationalism with ethnolinguistic ones in mind and, therefore, fails to apply this view of linguistic nationalism to the French nation as well (although he suggests the applicability of (ethno)linguistic nationalism to patois or dialects in France). This is probably because he wrote the manuscript against the background of the many newly independent nation-states that emerged in Central and Eastern Europe (especially in the ex-empery of the Habsburg Empire) after World War I, and was thus focused on them. Furthermore, Mauss even argues in favor of reducing the number of languages so that humanity will eventually acquire a universal (inter*national*) language, or will at least use the language of a major nation, for greater mutual understanding (Mauss [2013] 2018, pp. 147–148).

19 Two years after the amendment, the so-called Toubon Law, which aimed at the protection of French, was adopted in 1994.

20 This should never be understood as an excuse for terrorism. Bernard Maris, the economist and journalist who wrote one of the epigraphs to this article, was killed in the attacks on January 7, 2015. I express my sincerest condolences to the victims.
